# DynaLiRD: A dataset for dynamic line rating of overhead transmission lines, utilizing meteorological data and grid parameters based on the IEEE 738–2012 standard

**DOI:** 10.1016/j.dib.2025.112065

**Published:** 2025-09-15

**Authors:** Najmul Alam, M.A. Rahman, Md. Rashidul Islam

**Affiliations:** Department of Electrical & Electronic Engineering, Rajshahi University of Engineering & Technology, Kazla, Rajshahi 6204, Bangladesh

**Keywords:** Dynamic line rating, Dynamic thermal rating, Meteorological data, Transmission Line, IEEE 728–2012

## Abstract

This article presents DynaLiRD, a comprehensive dataset for dynamic line rating (DLR) of the Trang-Thap Cham 220 kV overhead transmission line. The DLR values are computed using the IEEE 738–2012 standard based on historical meteorological data such as ambient temperature, wind speed and direction, and global horizontal irradiance as well as detailed line parameters including conductor type, diameter, length, and elevation. To enhance the dataset’s applicability in cybersecurity and machine learning research, adversarially perturbed data is included using the fast gradient sign method (FGSM) and basic iterative method (BIM) under varying perturbation intensities. This dataset is essential for DLR estimation, dynamic thermal rating (DTR) forecasting, renewable energy integration into the grid, machine learning (ML) applications, infrastructure planning, energy policy development, and cybersecurity vulnerability investigation. Its structured format and inclusion of both clean and adversarial data make it valuable for evaluating the resilience of data-driven energy systems.

Specifications Table

**Table:** 

Subject	Energy (General)
Specific subject area	Dynamic line rating (DLR) of the overhead transmission line (OHL).
Type of data	*Table (CSV)*
Data collection	Weather data was sourced from the open-access platform https://energydata.info, providing hourly measurements of ambient temperature, wind speed, wind direction, and global horizontal irradiance (GHI) from January 1, 2020, to June 30, 2021
Data source location	Trang-Thap Cham 220 kV overhead line (Latitude: 11.758° N Longitude: 109.020° E)
Data accessibility	Github Repository name: Adversarial-attack-on-DLR Data identification number: 10.17632/xrhwdj7m7z.3 Direct URL to data: https://data.mendeley.com/datasets/xrhwdj7m7z/3
Related research article	https://doi.org/10.1016/j.epsr.2024.111289

## 1 Value of the Data

• The dataset provides critical data on dynamic line rating (DLR) calculations for overhead lines, calculated using IEEE 738–2012, facilitating applications in smart grid management and optimization of power transmission lines.
• It supports efficient grid management by supplying real-time DLR values, essential for integrating variable renewable energy sources such as wind and solar into the grid.
• The data aids in evaluating the thermal limits of transmission lines, contributing to both short-term and long-term planning of energy infrastructure.
• It offers a basis for DLR research, enhancing the use of existing transmission lines under varying environmental conditions and enabling DLR or dynamic thermal rating (DTR) forecasting.
• The dataset is applicable across disciplines, including energy policy, smart grids, machine learning, and cybersecurity, enabling a broad range of research applications.

## 2 Background

The motivation behind compiling this dataset stems from the need for accurate DLR values to optimize the operation of overhead lines (OHLs) in smart grids [1]. DLR is critical for determining the real-time thermal capacity of transmission lines, ensuring efficient energy flow while preventing overloading. The dataset was generated using the IEEE 738–2012 standard, which provides a method for calculating the thermal rating of OHLs based on environmental factors and conductor properties [2]. This dataset is essential for accurately assessing the capacity of lines under varying conditions, as traditional static ratings often fail to account for changing environmental variables. This dataset also includes adversarially attacked data generated using the fast gradient sign method (FGSM) and basic iterative method (BIM), which is essential for analyzing the adversarial impact on machine learning (ML) models and its potential implications for energy infrastructure.

## 3 Data Description

**Fig. 1:**
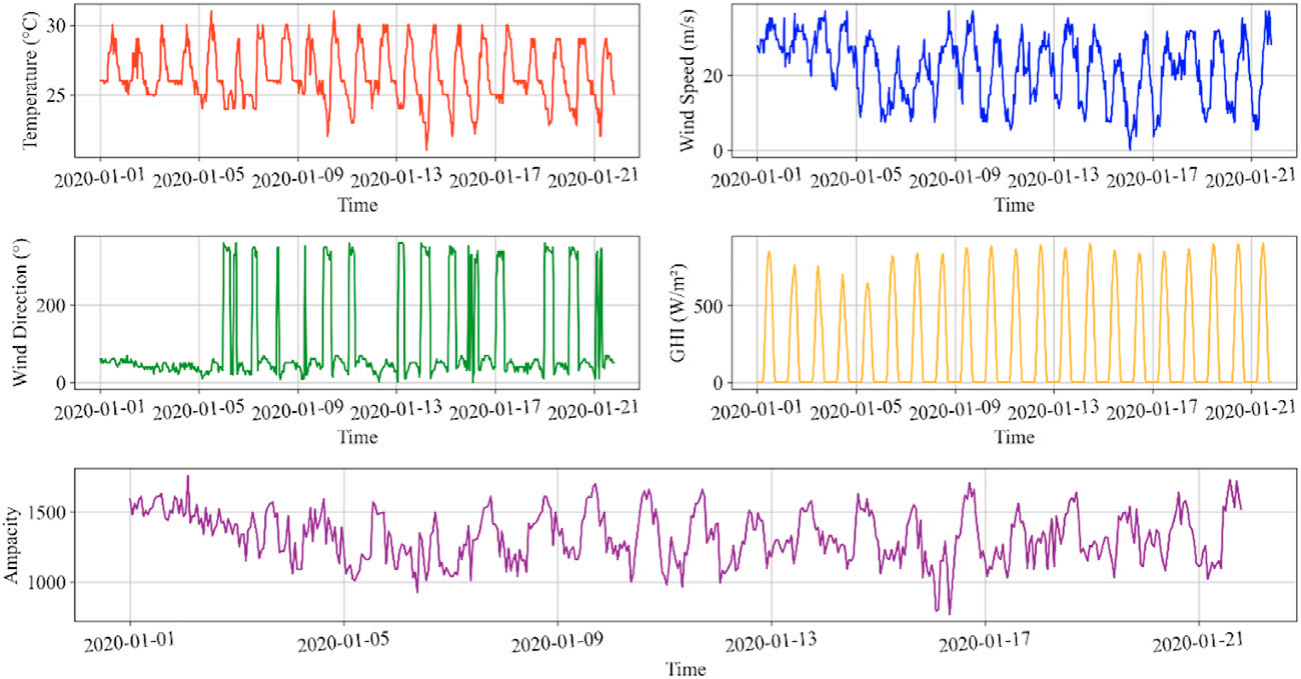
Visualization of the first 500 data points in the dataset, showcasing meteorological parameters including temperature, wind speed, wind direction, GHI, and line rating.

The dataset includes hourly meteorological data recorded from January 1, 2020, to June 30, 2021, alongside the DLR of the Trang-Thap Cham 220 kV overhead transmission line in Vietnam. Covering a period of 18 months, the dataset comprises a total of 13,128 data points [3]. The meteorological data consists of ambient temperature, wind speed, wind direction, and global horizontal irradiance (GHI). Table 1 shows the dataset column name and their description. Additionally Fig. 1 provides a visual representation of the dataset variables for the initial 500 data points. Furthermore, the test data has been attacked using FGSM and BIM, and the resulting adversarial examples with varying attack intensities and perturbation levels (ɛ) are also included.

**Table 1:** Dataset variables and their brief description.

Column name	Description
datetime	Timestamp of the recorded data (YYYY-MM-DD HH:MM format)
temp	Ambient temperature (°C)
Wind1	Wind speed (ms^-1^)
WinDir	Wind direction (°)
GHI	Global Horizontal Irradiance (Wm^-^²)
Ampacity	Line rating of the line

**Fig. 2:**
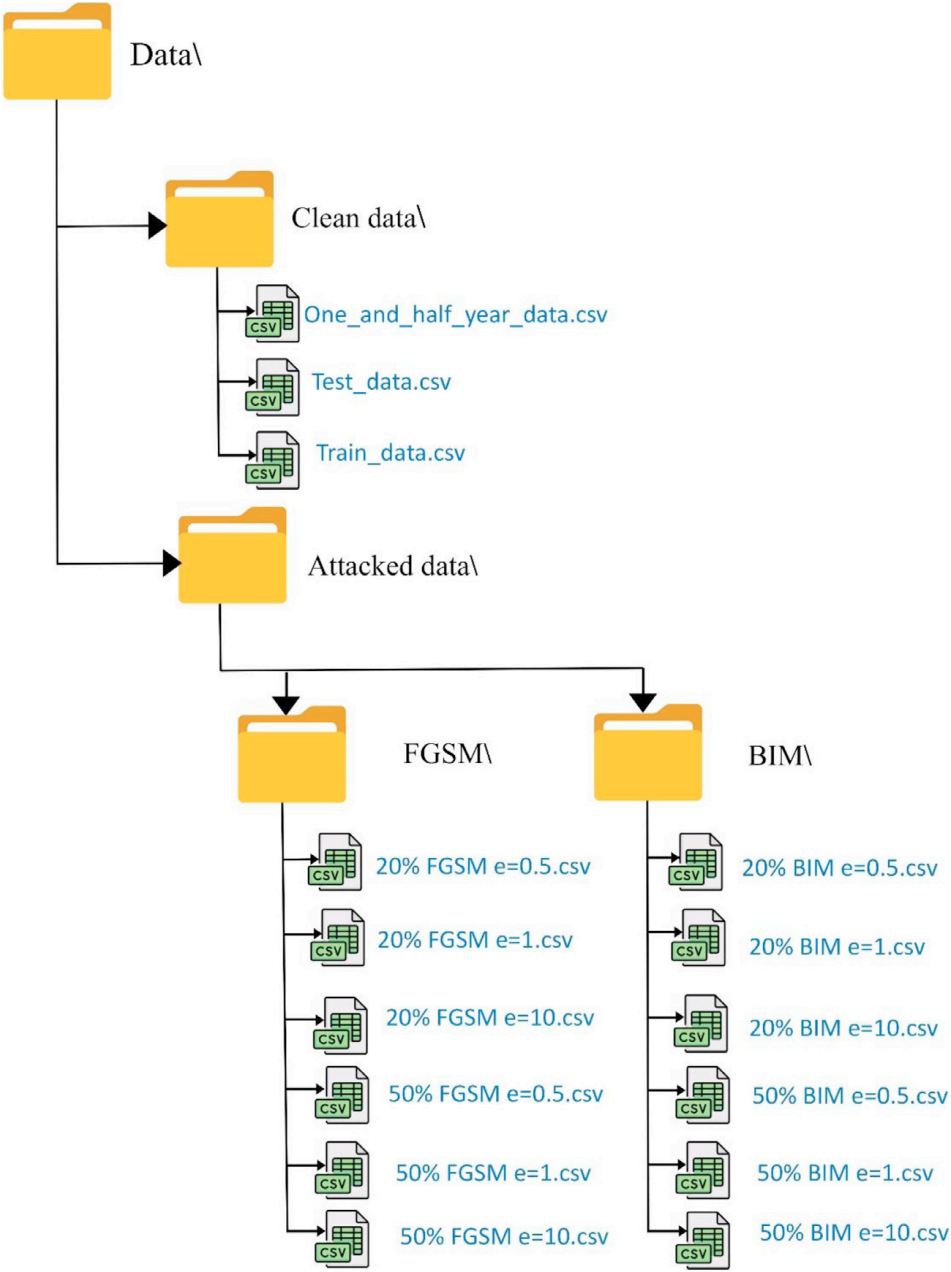
File organization of the dataset.

The dataset is structured into two main segments: Clean Data and Attacked Data, as shown in Fig. 2. The Clean Data includes One_and_half_year_data.csv, containing historical records for DLR calculations, along with Train_data.csv and Test_data.csv for model training and evaluation, respectively. The Attacked Data is divided into two adversarial attack types: FGSM and BIM. Each attack is performed on the temperature column with perturbation levels (ɛ) of 0.5, 1, and 10, affecting either 20 % or 50 % of the dataset, resulting in six variations for both FGSM and BIM. This dataset enables the evaluation of adversarial robustness in DLR, supporting research on cybersecurity vulnerabilities in energy infrastructure.

## 4 Experimental Design, Materials and Methods

This section provides an overview of IEEE 738–2012, FGSM, and BIM, which are used to generate the dataset. IEEE 738–2012 defines the standards for calculating the current-carrying capacity of overhead conductors, while FGSM and BIM are adversarial techniques employed to create perturbations for testing machine learning model robustness.

### 4.1 IEEE 738–2012

The DLR in this dataset is calculated using the IEEE 738–2012 standard. To compute the DLR, both meteorological data and line parameters are required [4]. Fig. 1 illustrates the metrological parameters, while Table 2 presents the OHL parameters employed for DLR calculation. This section also provides a brief overview of the IEEE 738–2012 standard, which has been utilized for the calculation of the DLR.

**Table 2:** Parameters and corresponding values for the Trang-Thap Cham 220 kV overhead transmission line.

Parameter	Value	Unit
Line length	117.794	Km
Nominal voltage	220	kV
Conductor type	ACSR	
Conductor diameter (D)	27.5	mm
Conductor sections	394/51.1	mm^2^
Absorbability	0.9	
Emissivity (ɛ)	0.7	
Static line rating	850	A
Elevation	30–35	m
Latitude	11.758° N	
Longitude	109.020° E	
Solar absorptivity (α)	0.8	
Conductor's surface area (A)	0.0864	m^2^/meter
AC resistance at the average conductor temperature; R(T_avg_)	1.4085 × 10^–4^	Ω/m

**Fig. 3:**
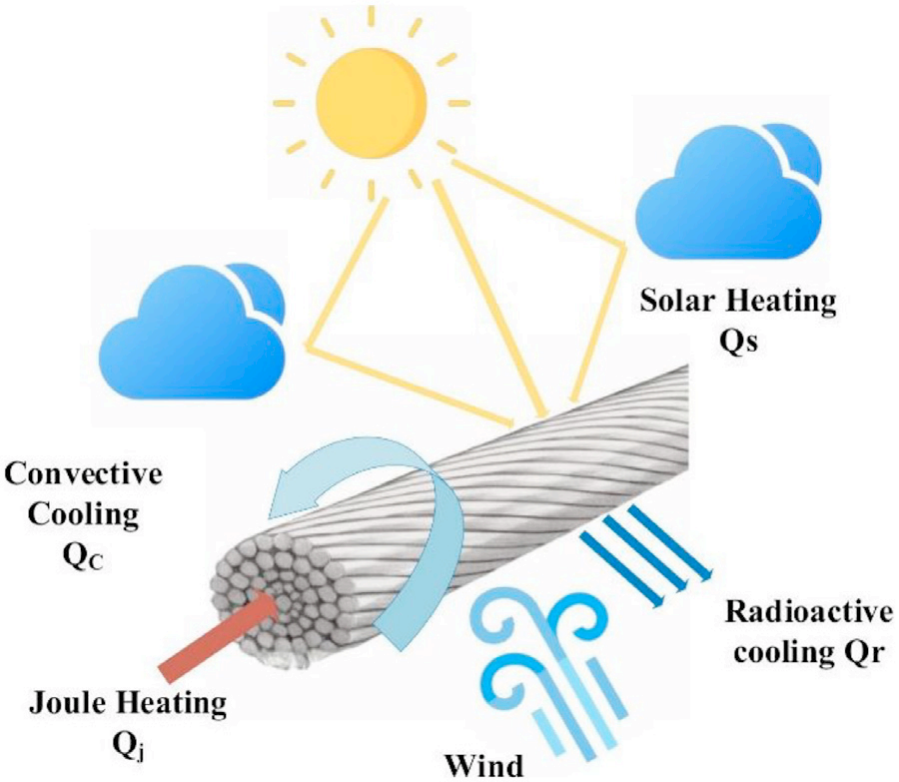
The thermal equilibrium process of OHLs [1].

The calculation of DLR using IEEE 738–2012 is based on a thermal balance equation that accounts for the heat dissipated and gained by an overhead conductor under varying environmental conditions [5]. Fig. 3 is the illustration of the heat balance of the OHLs.

The fundamental equation governing this balance is expressed as:

(1) $$Q_{r}+Q_{c}=Q_{j}+Q_{s}$$

where Q_r_ represents radiated heat loss, Q_c_ denotes convective heat loss, Q_j_ corresponds to Joule heating, and Q_s_ accounts for heat gain from solar radiation. Eq. (1) ensures that the conductor temperature remains within permissible operational limits by maintaining equilibrium between the heat dissipated and the heat absorbed.

The radiated heat loss, Q_r_, follows the Stefan-Boltzmann law and is given by:

(2) $$Q_{r}=\sigma \times \varepsilon \times A\times \left({T_{c}}^{4}-{T_{a}}^{4}\right)$$

where σ is the Stefan-Boltzmann constant, ε is the emissivity of the conductor, A is the conductor's surface area per unit length, T_c_ is the conductor surface temperature, and T_a_ is the ambient temperature. Convective heat loss, Q_c_, is composed of natural convection, expressed as:

(3) $${Q_{c}}^{n}=3.3645\times {\rho_{f}}^{0.5}\times D^{0.75}\times {\left(T_{c}-T_{a}\right)}^{1.25}$$

and forced convection, which depends on wind conditions. At low wind speeds, forced convection follows:

(4) $${Q_{c}}^{f}=K_{\text{angle}}\times K_{f}\times \left[1.01+1.13\times {N_{\text{Re}}}^{0.52}\right]\times \left(T_{c}-T_{a}\right)$$

while at higher wind speeds, it is expressed as:

(5) $${Q_{c}}^{f}=K_{\text{angle}}\times K_{f}\times 0.754\times {N_{\text{Re}}}^{0.6}\times \left(T_{c}-T_{a}\right)$$

where K_f_ is the thermal conductivity of air as a function of film temperature T_f_, and K_angle_ is the wind direction factor given by:

(6) $$K_{\text{angle}}=1.194-\text{sin}\left(\beta \right)-0.194\times \text{cos}\left(2\beta \right)+0.368\times \text{sin}\left(2\beta \right)$$

Joule heating, Q_j_, which represents resistive heating due to the electrical current, is computed as:

(7) $$Q_{j}=I^{2}\times R\left(T_{\text{avg}}\right)$$

where I is the conductor current and R(T_avg_) is the AC resistance at the average conductor temperature. Solar heat gain, Q_s_, is dependent on solar irradiance and is formulated as:

(8) $$Q_{s}=\alpha \times G\times A^{\prime }\times \text{sin}\left(\theta \right)$$

where α is the absorptivity of the conductor, G is the incident solar radiation, A is the projected conductor area per unit length, and θ is the effective angle of solar incidence.

By solving the thermal balance Eq. (1), the dynamic line rating can be determined as:

(9) $$\;\;I=\sqrt{\frac{Qr+Qc-Qs}{R.\left(Tavg\right)}}$$

which provides an adaptive ampacity value considering real-time variations in environmental parameters. The IEEE 738–2012 standard further incorporates transient thermal modeling to account for time-dependent variations, where time-step selection ensures that conductor temperature changes are accurately captured. This approach enables precise estimation of dynamic ampacity, optimizing the transmission capacity while maintaining safe operational limits in modern power systems.

### 4.2 Fast gradient sign method (FGSM)

The FGSM is an adversarial attack technique used to generate perturbed inputs that mislead machine learning models [6]. It modifies the original input X by adding a small perturbation in the direction of the gradient of the loss function with respect to the input. The equation for FGSM is:

(10) $$X_{\text{adv}}=X{+}_{\varepsilon }\cdot \text{sign}\left(\nabla_{X}J\left(X,y\right)\right)$$

Here, ɛ controls the magnitude of the perturbation, and the gradient ∇_X_J(X,y) helps determine the direction of the attack. This method is effective in testing model robustness against adversarial examples.

### 4.3 The basic iterative method (BIM)

BIM is an extension of the FGSM that applies multiple iterations of perturbations to generate stronger adversarial examples. In each iteration, BIM adds a small perturbation based on the gradient of the loss function and the sign of the gradient, refining the adversarial example [7]. The equation for BIM is:

(11) $${X_{\text{adv}}}^{k+1}=\text{cli}p_{X,\varepsilon }\left({X_{\text{adv}}}^{k}+\varepsilon \cdot \text{sign}\left(\nabla_{X}J\left({X_{\text{adv}}}^{k},y\right)\right)\right)$$

Here, ɛ is the step size, and clip_X,ϵ_​ ensures the adversarial example stays within a valid range, typically [*X*−ɛ, *X*+ɛ]. BIM is more powerful than FGSM due to its iterative approach, making it more effective in testing the robustness of machine learning models against adversarial attacks.

## 5 Discussion On Standard Update

The DLR values in this dataset were calculated using the IEEE 738–2012 standard, which provides thermal modelling guidelines for overhead conductors based on environmental and physical parameters. In 2023, the standard was updated to IEEE 738–2023, introducing several enhancements, including refined convective heat transfer models, improved treatment of solar heating and radiative losses, and expanded annexes offering guidance on weather data selection and parameter sensitivity. Despite these updates, the core thermal balance methodology remains fundamentally unchanged. As such, while IEEE 738–2023 enables more accurate and flexible modelling, especially under dynamic and real-time conditions, the differences are not expected to significantly affect the validity or utility of the dataset for research and benchmarking purposes. The dataset still offers reliable results under the IEEE 738–2012 framework and can be readily adapted to incorporate the refinements of the newer standard if required.

## Limitations

The dataset represents a single transmission line (Trang–Thap Cham 220 kV), which may limit direct generalization to other line types or geographical regions; however, the methodology used is adaptable and can be extended to other contexts. DLR is calculated using the IEEE 738–2012 standard, while the more recent IEEE 738–2023 standard introduces additional modeling refinements that are not reflected in this version. Though the core thermal modeling principles remain consistent across both standards.

## Ethics statement

The authors declare that this research did not involve human or animal subjects, nor did it include any data collection from social media platforms.

## Credit author statement

**Najmul Alam:** Conceptualization, Data curation, Formal analysis, Investigation, Methodology, Resources, Software, Visualization, Writing – original draft; **M. A. Rahman:** Conceptualization, Methodology, Project administration, Writing – original draft; **Md. Rashidul Islam:** Validation, Supervision, Writing – review and editing.

## Data Availability

Mendeley DataDynaLiRD: A dataset for dynamic line rating of overhead transmission lines, utilizing meteorological data and grid parameters based on the IEEE 738-2012 standard (Original data)
